# Greater muscle electrode distance and fat mass affect motor units identification from high-density surface EMG in the vastus lateralis muscle

**DOI:** 10.1038/s41598-025-24966-w

**Published:** 2025-11-20

**Authors:** Alessandro Sampieri, Gioi Spinello, Martino V. Franchi, Francesco Campa, Antonio Paoli, Tatiana Moro, Andrea Casolo

**Affiliations:** 1 Department of Biomedical Sciences, University of Padova, Padua, 35131 Italy; 2 CIR-MYO Myology Centre, University of Padova, Padua, Italy

**Keywords:** Volume conductor, Subcutaneous fat, Muscle-electrode distance, Electromyography, Decomposition, Motor unit, Physiology, Neurophysiology, Motor control, Motor neuron

## Abstract

Decomposing high-density surface electromyography (HDsEMG) signals enables non-invasive identification of motor units (MU); however, anatomical factors contribute to variability in the identified MU number across individuals. This study examined the influence of fat mass (FM%), muscle-electrode distance (MED), and muscle size on MU identification in the vastus lateralis. Thirty-three young (19–30 years) and twenty-eight older adults (66–82 years), including both sexes, performed isometric knee extensions at 15%, 35%, 50%, and 70% of maximal voluntary force (MVF) while HDsEMG signals were recorded. Whole-body and regional FM% were assessed using bioelectrical impedance analysis (BIA) and dual-energy X-ray absorptiometry (DXA), while MED and muscle size were measured with ultrasonography. Females exhibited greater MED and FM%, resulting in fewer identified MU than males. Significant negative correlations were found between the identified MU number and whole-body FM% (15%-70% MVF: BIA: r_s_=-0.508 to r_s_=-0.415; DXA: r_s_=-0.531 to r_s_=-0.337), leg FM% (15%-70% MVF: r_s_=-0.579 to r_s_=-0.582), thigh FM% (15%-70% MVF: r_s_=-0.614 to r_s_=-0.620), and MED (15%-70% MVF: r_s_=-0.581 to r_s_=-0.670). Notably, segmented regression analysis revealed a threshold at ~ 0.7 cm MED, below which a greater number of MU could be identified. Our findings highlight the negative impact of high FM% and MED on MU identification from HDsEMG decomposition.

## Introduction

Voluntary muscle contraction is driven by motor units (MU), which translate the synaptic input into muscle-generated forces^1,2^. The compound electrical activity at the muscle fiber level, corresponding to MU action potentials, can be recorded via high-density surface electromyography (HDsEMG). Combined with decomposition methods, such as Blind Source Separation, HDsEMG enables the assessment of large populations of active MU during voluntary movements^3–5^.

While the validity and reliability of the decomposition methods for signals recorded during isometric contractions are well-established^6–8^, various factors influence the quality of the decomposition outcome. This leads to greater variability in the number of identified MU across individuals, force levels, and muscles^9^. In this context, a key factor is the volume conductor, which represents the nonhomogeneous medium through which electrical signals propagate from their source (i.e., the muscle fibers) to the surface (i.e., recording electrodes). It includes biological tissues such as the skin, connective tissue, and subcutaneous fat^10,11^. Among these, subcutaneous fat functions as a low-pass filter due to different conductivity properties compared to skeletal muscle^11,12^, reducing the spatial and frequency bandwidth of MU action potentials detected at the skin’s surface, and causing overlap of waveforms from multiple MU^10,13,14^. Therefore, increased muscle-electrode distance (MED) (i.e., the distance between the recording electrodes and the source of MU action potentials) decreases the amplitude of detected signals and complicates the decomposition process due to the similarity of MU waveform^10^. Previous research indicates that greater MED is associated with a reduced number of identified MU from the biceps brachii in a cohort of healthy male individuals^15^. By contrast, the influence of muscle size on MU identification remains less clear, with conflicting results reported in the literature^15,16^.

Over the past two decades, the study of MU properties has expanded to applications in rehabilitation^17^, prosthetic control^18^, disuse^19,20^ and exercise training^21–23^. However, large variability in the number of identified MU poses challenges for experimental studies, particularly in longitudinal research (e.g., training interventions) or cross-sectional comparisons, where a MU population-level perspective is essential to draw reliable inferences. Thus, exploring individual features that influence HDsEMG signal decomposition remains a priority. Practical, time-efficient, and non-invasive approaches may offer a means to identify morphological characteristics that facilitate the detection of a representative number of MU.

Body composition assessment, in particular, can provide valuable insights into anatomical features, such as fat mass distribution, that affect HDsEMG signals and the properties of the volume conductor. To address this, in the present study we employed multiple body composition assessment methods including bioelectrical impedance analysis (BIA) to estimate relative whole body fat mass (FM%), Dual-energy X-ray Absorptiometry (DXA) to estimate both whole-body and regional (i.e., right leg and thigh) FM% and lean soft tissue (i.e., fat-free mass excluding bone mass), and b-mode ultrasound (US) to measure muscle size and MED.

Based on these assessments, our aim was to determine the influence of FM%, MED, and muscle size on the number of identified MU in the vastus lateralis (VL) muscle during submaximal voluntary isometric knee extension. We recruited a heterogeneous population of healthy young and older adults, including both males and females, to account for inter-individual variability in force and body composition characteristics. Specifically, females typically present higher levels of peripheral adiposity and subcutaneous fat compared to males^24^, while older adults generally show a decline in lean soft tissue, increased infiltration of FM% in muscles, and reduced appendicular fat^25^. We hypothesized that higher FM% and greater MED would negatively affect decomposition outcomes, leading to a reduced number of detectable MU. Additionally, we hypothesized that muscle size would either have no significant influence on MU identification or affect it only at low intensities, consistent with previous findings^15^.

## Results

### Anthropometric, body composition, physical activity, and muscle force and size characteristics

Tables 1 and 2 summarize participants’ characteristics, categorized by age and sex, respectively. Overall, young adults exhibited lower BMI and FM%, but greater quadriceps and VL CSA, and maximal voluntary strength (i.e., maximal voluntary force (MVF) and maximal voluntary torque (MVT) compared with older adults. Moreover, despite their smaller representation in the sample (*n* = 15), females exhibited higher FM% and MED, but lower CSA and maximal voluntary strength than males, regardless of age. The observed groups differences (young vs. older adults and females vs. males), highlighted throughout these comparisons, contributed to the overall variability in FM%, MED, muscle size, and maximal strength across the cohort.

**Table 1:** Anthropometric, body composition, physical activity and muscle strength characteristics of the participants by age group.

	Group		*p*-value
	Y (*N* = 33)	O (*N* = 28)	
**Age (yr.)**	23.58 ± 2.91	71.18 ± 4.95	< 0.001
**Gender (n)**	M = 24; F = 9	M = 22; F = 6	-
**BMI (kg·m** ^**−2**^ **)**	22.51 ± 2.49	25.95 ± 3.40	< 0.001
**WHOLE-BODY FM DXA (%)**	23.94 ± 5.1	32.03 ± 5.90	< 0.001
**FM RIGHT LEG DXA (%)**	26.53 ± 7.07	31.4 ± 8.36	< 0.001
**FM RIGHT THIGH DXA (%)**	25.89 ± 6.61	30.15 ± 8.09	< 0.001
**WHOLE-BODY FM BIA (%)**	22.43 ± 7.71	30.49 ± 7.62	0.003
**QUADRICEPS CSA (cm** ^**2**^ **)**	68.38 ± 14.79	53.07 ± 13.71	0.001
**VL CSA (cm** ^**2**^**)**	23.12 ± 5.02	17.89 ± 4.56	< 0.001
**MT (cm)**	2.54 ± 0.39	2.48 ± 0.41	0.721
**MED (cm)**	0.68 ± 0.23	0.69 ± 0.31	0.987
**GPAQ (MET·wk** ^**−1**^ **)**	2607.97 ± 1731.48	2069.78 ± 1407.88	0.298
**MVF (N)**	656.98 ± 164.03	497.47 ± 135.12	< 0.001
**MVT (N·m)**	227.93 ± 60.65	171.73 ± 48.85	< 0.001

Data are presented as mean ± SD. Between-group comparisons were performed with the Mann-Whitney U test. BIA: Bioelectrical Impedance Analysis; BMI: Body mass index; CSA: Cross-sectional area; DXA: Dual-Energy X-ray Absorptiometry; F: Females; FM: Fat mass; GPAQ: Global physical activity questionnaire; M: Males; MT: Muscle thickness; MVF: Maximal voluntary force; MVT: Maximal voluntary torque; O: Older adults; VL: Vastus lateralis; Y: Young adults.

**Table 2:** Anthropometric, body composition, physical activity and muscle strength characteristics of the participants by gender.

	Group		*p*-value
	Females (*N* = 15)	Males (*N* = 46)	
**BMI (kg·m** ^**−2**^ **)**	22.35 ± 1.16	24.66 ± 3.69	0.014
**WHOLE-BODY FM DXA (%)**	32.15 ± 5.74	26.19 ± 6.5	0.004
**RIGHT LEG FM DXA (%)**	38.23 ± 7.04	25.69 ± 5.52	< 0.001
**RIGHT THIGH FM DXA (%)**	36.53 ± 6.63	24.86 ± 5.22	< 0.001
**WHOLE-BODY FM BIA (%)**	33.54 ± 4.18	23.71 ± 8.32	< 0.001
**QUADRICEPS CSA (cm** ^**2**^ **)**	39.95 ± 9.37	64.85 ± 14.00	< 0.001
**VL CSA (cm** ^**2**^ **)**	14.19 ± 2.95	21.77 ± 4.97	< 0.001
**MT (cm)**	2.25 ± 0.31	2.57 ± 0.39	0.022
**MED (cm)**	1.07 ± 0.22	0.59 ± 0.18	< 0.001
**GPAQ (MET·wk** ^**−1**^ **)**	3062.58 ± 1567.80	2209.47 ± 1613.26	0.128
**MVF (N)**	455.95 ± 146.34	624.54 ± 158.47	< 0.001
**MVT (N·m)**	155.77 ± 57.79	216.91 ± 56.39	< 0.001

Data are presented as mean ± SD. Between-group comparisons were performed with the Mann-Whitney U test. BIA: Bioelectrical Impedance Analysis; BMI: Body mass index; CSA: Cross-sectional area; DXA: Dual-Energy X-ray Absorptiometry; F: Females; FM: Fat mass; GPAQ: Global physical activity questionnaire; M: Males; MT: Muscle thickness; MVF: Maximal voluntary force; MVT: Maximal voluntary torque; O: Older adults; VL: Vastus lateralis; Y: Young adults.

### Number of MU across participants and target forces

After HDsEMG signals decomposition, a total of 1890 unique MU were identified from the VL across all participants and target forces (15%, 35%, 50%, and 70% MVF).

**Fig. 1:**
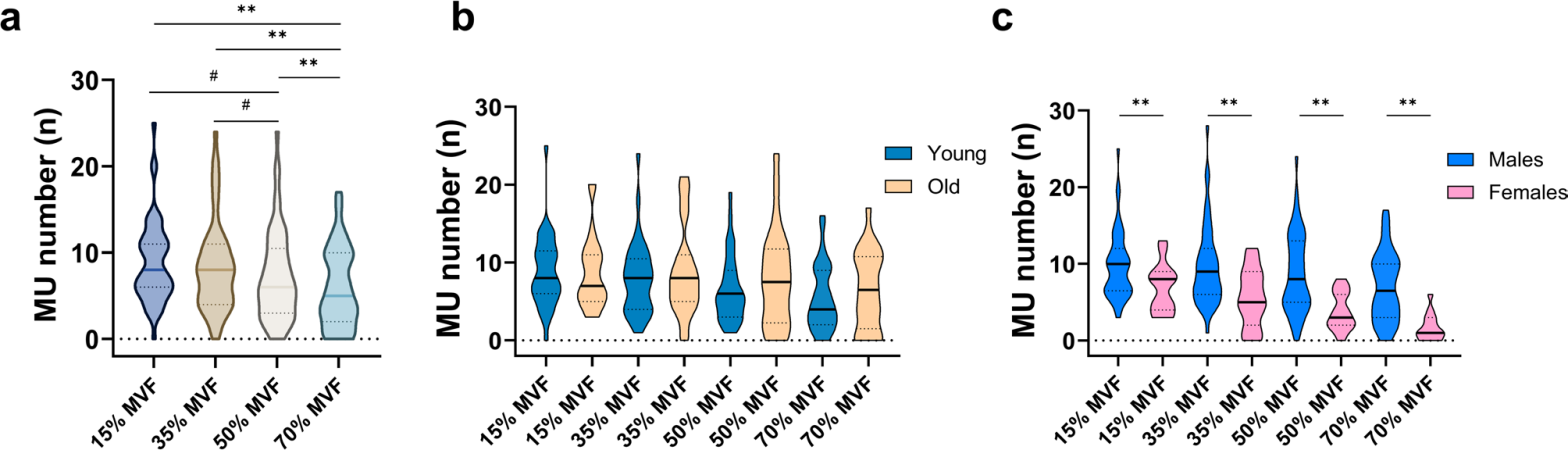
Violin plots of the number of identified MU across target forces. **(a)** Distribution of MU for the entire cohort. The Friedman test revealed significant differences between 70% MVF and 50%, 35%, and 15% MVF (**, *p* < 0.001), and between 50% MVF and 35% and 15% MVF (#, *p* < 0.05). **(b)** Distribution of identified MU across target forces, grouped by age. No significant differences were observed between young and older adults. **(c)** Distribution of MU across target forces, grouped by sex. Females exhibited a consistently lower number of identified MU at all target forces (**, *p* < 0.001). Within each graph, the dashed lines represent the 25th and 75th percentile, while the black line indicates the median value.

A significant main effect of target force on MU number was observed across the entire cohort of participants (*p* < 0.001) (Fig. 1, a). Post-hoc comparisons revealed significant differences in the number of MU between 70% MVF and 15%, 35%, and 50% (*p* < 0.001 in all cases). Further significant differences were found between 50% MVF and 15% and 35% MVF (*p* = 0.017 and *p* = 0.039, respectively). Thus, fewer MU were identified from the VL at higher forces, regardless of age or sex.

The average number of MU identified per participant did not differ between young and older adults (average across all target forces: 8 ± 5 and 8 ± 5 MU per participant, respectively). Specifically, the average number of MU per participant was 9 ± 4 and 9 ± 4 at 15% MVF, 8 ± 5 and 9 ± 5 at 35% MVF, 7 ± 4 and 8 ± 6 at 50% MVF, 5 ± 5 and 6 ± 5 at 70% MVF, for young and older adults, respectively (Fig. 1, b). Furthermore, females exhibited a significantly lower number of identifiable MU than males across all target forces (*p* < 0.05 in all cases). Specifically, the average number of MU per participant was 7 ± 3 and 10 ± 4 at 15% MVF, 6 ± 4 and 10 ± 5 at 35% MVF, 4 ± 3 and 9 ± 5 at 50% MVF, and 2 ± 2 and 7 ± 4 at 70% MVF, for females and males, respectively (Fig. 1, c).

### Relation between identified MU number, fat mass and muscle electrode distance

**Fig. 2:**
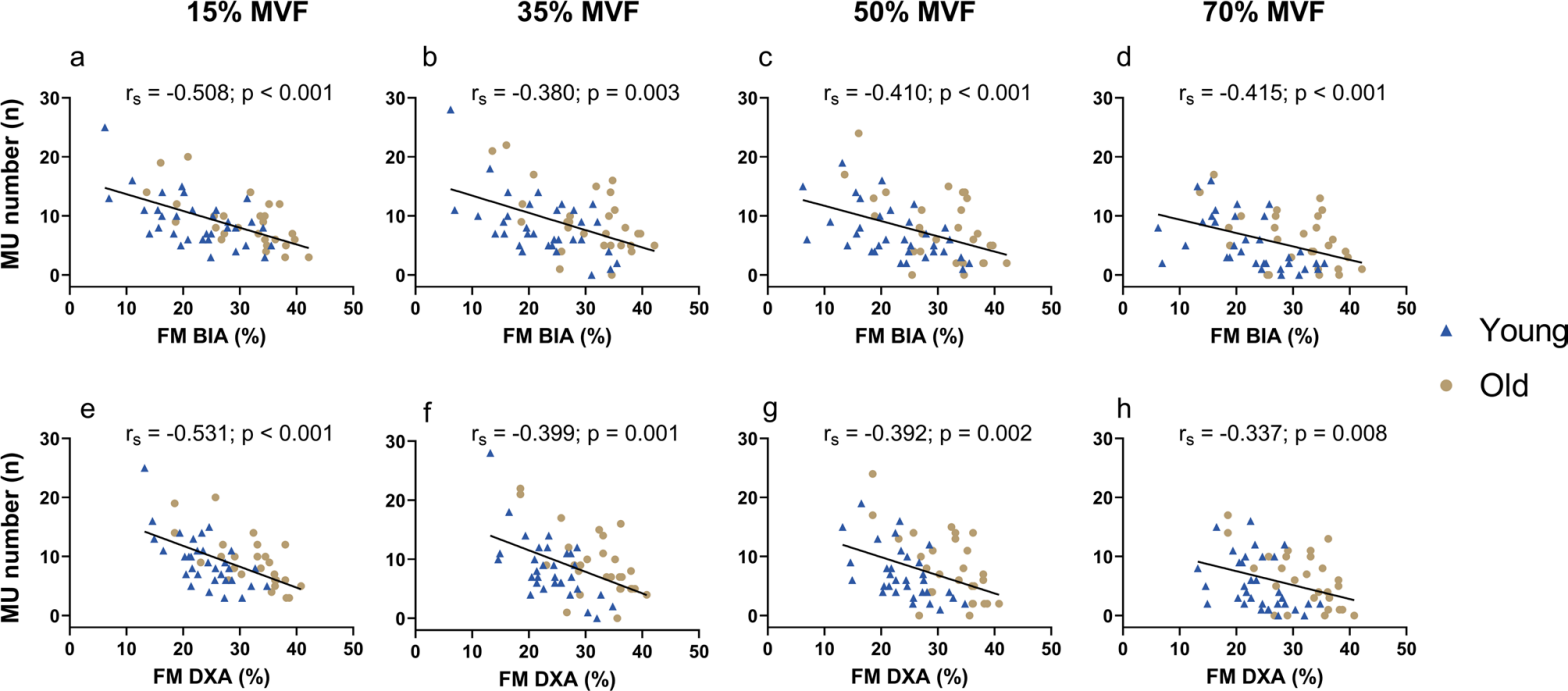
Correlation between whole-body FM% and the number of identified MU across all target forces. On the top, correlations between whole-body FM% estimated by BIA at 15% **(a)**, 35% **(b)**, 50% **(c)**, and 70% MVF **(d)**. On the bottom, correlations between whole-body FM% estimated by DXA at 15% **(e)**, 35% **(f)**, 50% **(g)**, and 70% MVF **(h)**. Each point within the plot represents the number of identified MU per participant. The black trendlines highlight the direction of the correlation. Specifically, the higher the FM%, the fewer the number of MU that can be accurately detected. FM: fat mass; BIA: bioelectrical impedance analysis; DXA: dual-energy X-ray absorptiometry; r_s_: Spearman correlation index; Y: young adults; O: older adults.

Considering the entire cohort of participants, FM% estimated by both BIA and DXA was negatively associated with the number of MU identified. Specifically, we found a significant negative correlation between whole-body FM% assessed by BIA and the number of MU identified at 15% (r_**s**_ = −0.508, *p* < 0.001), 35% (r_**s**_ = −0.380, *p* = 0.003), 50% (r_**s**_ = −0.410, *p* < 0.001), 70% MVF (r_**s**_ = −0.415, *p* < 0.001) (Fig. 2a, b, c, d). Similarly, whole-body FM% assessed by DXA was negatively associated with the number of MU identified at 15% (r_**s**_ = −0.531; *p* < 0.001), 35% (r_**s**_ = −0.399, *p* = 0.001), 50% (r_**s**_ = −0.392, *p* = 0.002), 70% MVF (r_**s**_ = −0.337, *p* = 0.008) (Fig. 2e, f, g, h).

**Fig. 3:**
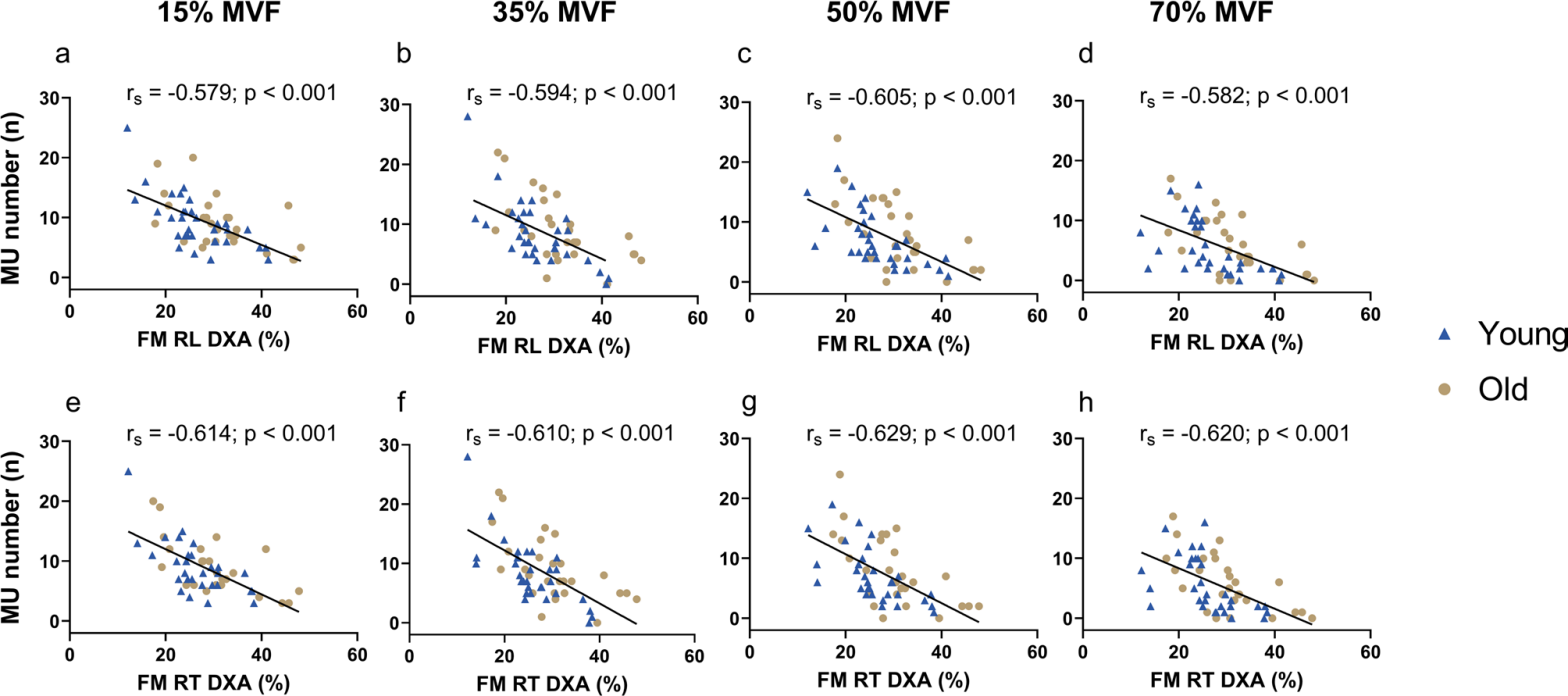
Correlation between FM% of the right leg and thigh and the number of identified MU across all target forces. On the top, the correlations between FM% of the right leg estimated by DXA at 15% **(a)**, 35% **(b)**, 50% **(c)**, and 70% MVF **(d)**. On the bottom, the correlations between FM% of the thigh estimated by DXA at 15% **(e)**, 35% **(f)**, 50% **(g)**, and 70% MVF **(h)**. Each point within the plot represents the number of identified MU per participant. The black trendline highlights the negative correlation between the two variables across all target forces. RL: right leg; RT: right thigh; DXA: dual-energy X-ray absorptiometry; r_s_: Spearman correlation index; Y: young adults; O: older adults.

When FM% of the right leg, assessed by DXA, was considered, significant negative correlations with the number of identified MU were observed at 15% (r_**s**_ = − 0.579, *p* < 0.001), 35% (r_**s**_ = −0.594, *p* < 0.001), 50% (r_**s**_ = −0.605, *p* < 0.001), and 70% MVF (r_**s**_ = −0.582, *p* < 0.001) (Fig. 3a, b, c, d). Similarly, FM% of the right tight was negatively correlated with the number of MU identified at 15% (r_**s**_ = − 0.614, *p* < 0.001), 35% (r_**s**_ = −0.610, *p* < 0.001), 50% (r_**s**_ = −0.629, *p* < 0.001), 70% MVF (r_**s**_ = −0.620, *p* < 0.001) (Fig. 3e, f, g, h*).*

**Fig. 4:**
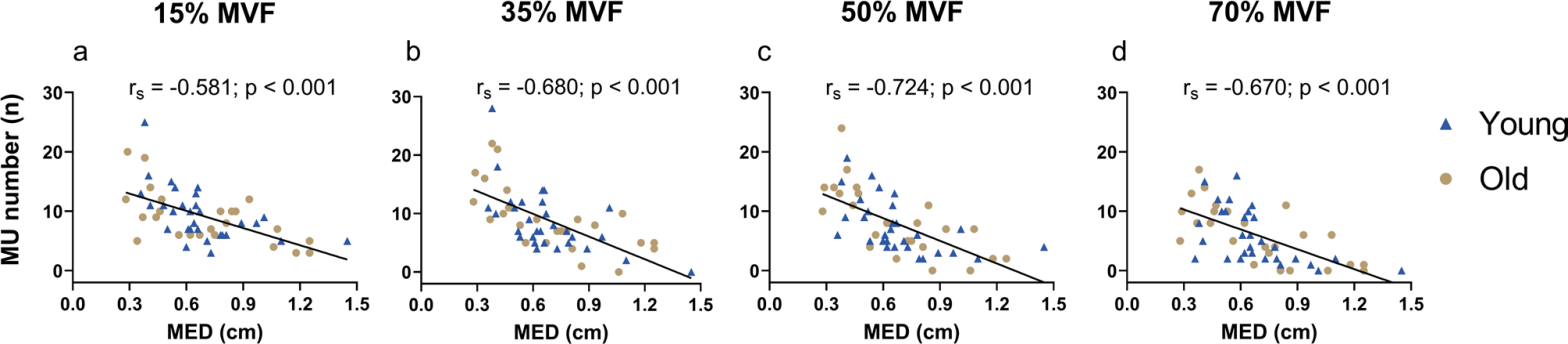
Correlation between MED and the number of identified MU across all target forces. Correlations between MED assessed by ultrasounds at 15% **(a)**, 35% **(b)**, 50% **(c)**, and 70% MVF **(d)**. Each point within the plot represents the number of identified MU per participant. The black trendline highlights the negative correlation between the two variables across all target forces. MED represents the average of three measurements taken 1 cm from the start, at the midpoint, and 1 cm before the end of the image. MED: muscle-electrode distance; r_s_: Spearman correlation index; Y: young adults; O: older adults.

The average MED was used as a more regional indicator of subcutaneous fat within the volume conductor. Significant negative correlations (*p* < 0.001 in all cases) were observed between MED and the number of MU identified at 15% (r_**s**_ = −0.581), 35% (r_**s**_ = −0.680), 50% (r_**s**_ = −0.724) and 70% MVF (r_**s**_ = −0.670) (Fig. 4a, b, c, d). In contrast, US-derived measures of muscle size (i.e., MT, quadriceps CSA and VL CSA) were not significantly associated with the number of identified MU (*p* > 0.05 in all cases).

**Fig. 5:**
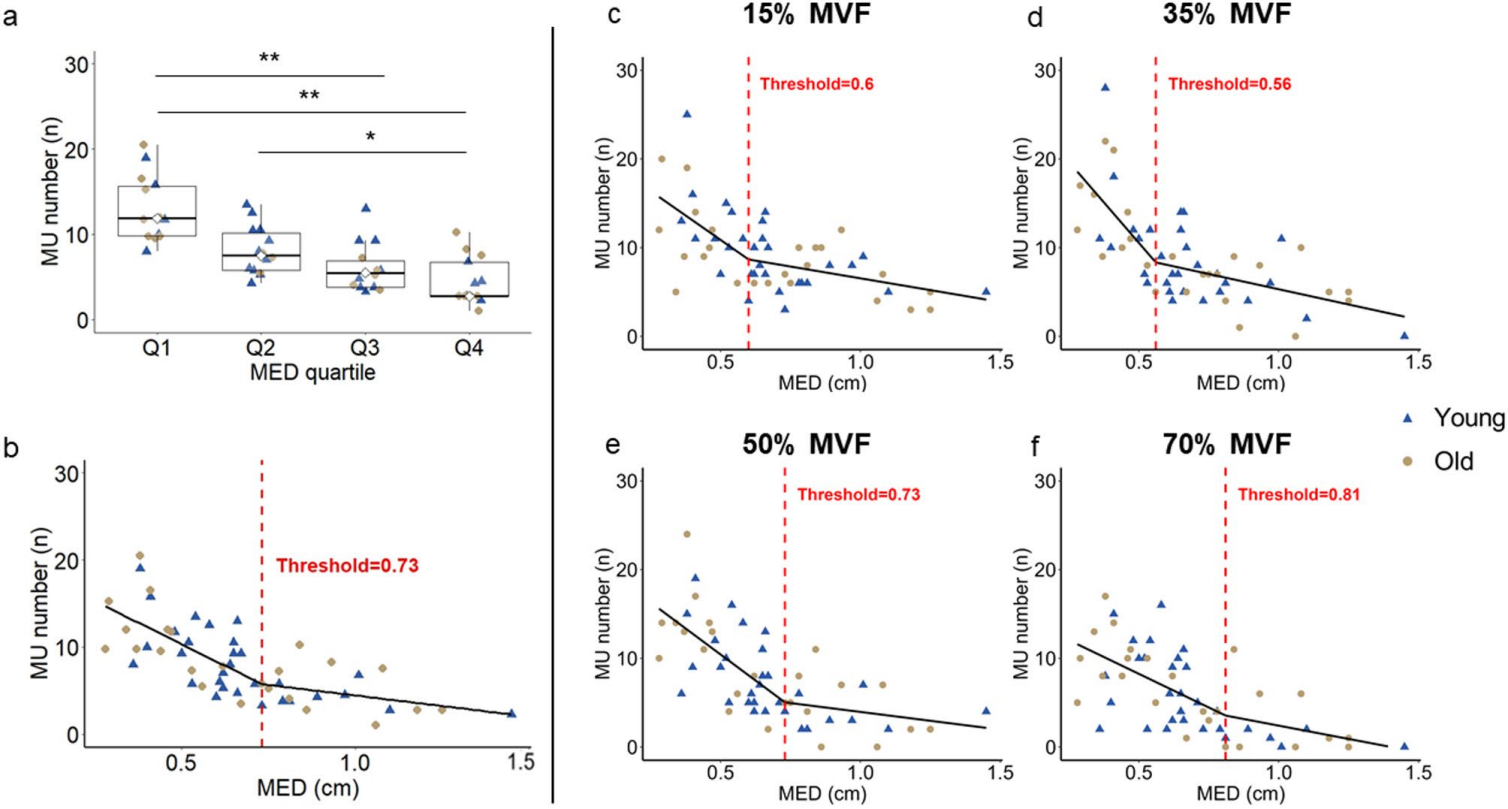
Quartile and segmental regression analysis and MED threshold. **(a)** Comparison of the number of identified MU across quartiles of MED. Boxplots show the distribution of MU number; the horizontal line represents the median, the box the interquartile range, and the whiskers the minimum and maximum values. Individual dots represent the average number of MU identified across all contractions. Segmented regression analysis between MED and the average number of identified MU across all contractions **(b)** and the analysis separated for each target force: 15% MVF **(c)**, 35% MVF **(d)**, 50% MVF **(e)**, and 70% MVF **(f)**. Solid black line indicates the fitted segmented regression, while the red dashed line marks the estimated thresholds. MED: muscle-electrode distance; *: p-value < 0.05; **: p-value < 0.001.

Since MED showed the strongest correlation with the number of identified MU, we further explored whether a specific threshold could be identified in this relationship, indicating the level of MED above which MU detection becomes limited. Participants were divided into quartiles based on MED values: Q1 (0.28–0.48 cm), Q2 (0.50–0.65 cm), Q3 (0.65–0.81 cm), and Q4 (0.84–1.45 cm). The average number of detected MU decreased progressively across quartiles (13 ± 4 in Q1 to 5 ± 3 in Q4), with significant group differences (*p* < 0.001). Post hoc comparisons showed that Q1 differed from Q3 (*p* < 0.001) and Q4 (*p* < 0.001), and Q2 differed from Q4 (*p* = 0.045; Fig. 5a). On average, individuals in the lower MED quartiles (Q1 and Q2; < 0.65 cm) exhibited ~ 11 detectable MU, whereas in the upper MED quartiles (Q3 and Q4; > 0.65 cm) ~ 5 detectable MU. Segmented regression analysis across all contraction levels identified a threshold at 0.73 cm MED. Specifically, below this value, MU detection decreased with increasing MED (slope = −19.9, 95% CI: − 27.6 to −12.1, *p* < 0.001), with an average of ~ 10 detected MU. Above the 0.73 cm threshold, no further significant decrease was observed (slope = −4.8, *p* = 0.180), with an average of ~ 5 MU (Fig. 5b). Consistent results were obtained at individual force levels, with thresholds ranging from 0.56 to 0.81 cm (Fig. 5c, d, e, f*).*

## Discussion

In this study, we investigated the association between body composition, anatomical features and the number of MU accurately decomposed from the VL muscle during isometric submaximal knee extensions. Our results demonstrate that FM% and MED negatively affected the outcome of HDsEMG signal decomposition, whereas muscle size, quantified by CSA and MT, showed no significant association.

Whole-body FM% estimated by both DXA and BIA showed similar correlations with the number of identified MU from VL, suggesting that higher FM% is associated with fewer identified MU. Importantly, when FM% was estimated at a more regional level, specifically in the anatomical region where the electrode grid was applied, the associations with MU detection became stronger. This likely reflects that regional FM% more directly captures the characteristics of the volume conductor that influence signal quality. Thus, for the VL muscle, assessing FM% at the level of the leg, or more precisely at the thigh when possible, may provide a better representation of the number of MU that can be reliably detected.

Consistent with these findings, greater MED was also strongly associated with fewer identified MU. MED represents a direct and more localized measure of the volume conductor size and subcutaneous fat thickness compared to estimates derived from regional FM% using DXA. In line with our results, a recent study^15^ reported a negative correlation between MED and the number of identified MU, though this effect was observed only up to 35% MVF in the biceps brachii muscle. In contrast, our results showed that MED significantly influenced MU detection even at higher force levels. This discrepancy may be attributed to the broader variability in MED observed in our sample, ranging from 0.28 to 1.45 cm (compared to 0.27 to 0.78 cm^15^ [unpublished data]), facilitated by the inclusion of both sexes and a wider age range. Additionally, it should be noted that the number of identified MU in our sample ranged from 0 to 24 MU at 50% MVF and 0–17 MU at 70% MVF, compared to 2–12 MU at 50% MVF and 0–10 MU at 70% MVF as reported in previous research^15^. Overall, regardless of force levels, previous evidence showed that increased MED is associated with greater subcutaneous fat, which acts as a spatial and temporal filter on EMG signals. This filtering effect reduces the amplitude of MU action potentials, thereby impairing their detection^10–12^. Supporting this, a study on individuals with obesity (BMI > 35 kg·m^−2^), who exhibited greater subcutaneous fat over VL measured with US (average: 1.82 ± 0.92 cm), reported lower EMG amplitude and altered spectral compared to healthy-weight controls^28^. These findings highlight the challenges in acquiring high-quality EMG signals from the VL in individuals with higher fat accumulation, particularly in the thigh region. Conversely, muscles with minimal overlying subcutaneous fat, such as tibialis anterior, facilitate the identification of a greater number of MU^9,29^. While this advantage allows the tibialis anterior to provide a more representative MU sampling, studying the quadriceps muscles remains essential for various applications, including investigating neural deficits following knee injuries or surgery^17^, disuse, or exploring the neural adaptations to resistance training targeting multi-joint exercises^19^.

Notably, our findings suggest, for the first time, a potential threshold of ~ 0.7 cm MED that may be considered in studies employing HDsEMG signal decomposition from VL. Quartile analysis indicated that participants with MED values < 0.65 cm (upper limit of Q2) showed, on average, ~ 11 detectable MU, whereas those with larger MED exhibited markedly fewer. Segmented regression analysis identified a threshold at 0.73 cm, below which an average of ~ 10 MU could be detected. These findings confirm the negative association between MED and MU number below the threshold of 0.73 cm. Conversely, it appears that when MED exceeds ~ 0.7 cm, additional factors may further limit MU detection. We would advise caution to interpret this ~ 0.7 cm MED threshold more as an indicative value, below which a higher number of MU can usually be identified from the VL in healthy individuals, rather than as a strict exclusion criterion. The decision to apply such a threshold should ultimately depend on the specific research question, experimental protocol, and the muscle of choice.

When participants with higher MED are included, larger sample sizes should be considered, exceeding those typically estimated by standard power analyses based solely on effect sizes. This is particularly relevant for longitudinal designs aimed at tracking MU over time, as only ~ 30% of MU can be reliably tracked over time^9^. Ensuring a sufficiently high number of detectable MU is therefore essential for representativeness and reliability. Future research should also prioritize HDsEMG decomposition algorithms, particularly in populations with greater MED or regional FM%. In this regard, the recently developed Swarm-Contrastive Decomposition algorithm has shown superior performance in both the number and accuracy of detected MU compared with traditional Blind Source Separation methods^30^. This approach dynamically adjusts the contrast function to the signal distribution, thereby improving the separation of highly similar MU action potentials, a condition frequently observed in recordings from deep muscles or those covered by thicker subcutaneous fat^30^.

Muscle size, quantified by CSA and MT measured under the electrode grid, showed no association with the number of identified MU across all target forces. These findings align with a previous simulation study reporting that muscle size does not influence the ability to distinguish individual MU action potentials^16^. By contrast, a recent study reported that a larger anatomical muscle area was associated with a greater number of detected MU, though only at low force levels^15^. It should be noted, however, that this study assessed CSA with magnetic resonance imaging (i.e., the gold standard technique for muscle size estimation^31^ and measured it directly beneath the electrode grid, whereas in the present study, quadriceps CSA was evaluated at 50% of the femur length. Moreover, this previous study^15^ focused on the biceps brachii, a fusiform muscle with fibers aligned longitudinally to the tendon^32^, in contrast to the pennate structure of the VL, where fibers are oriented obliquely relative to the tendon^32^. Such architectural differences may also contribute to the higher number of identifiable MU in the VL, as non-parallel (e.g., pennate) fiber arrangements provide greater discriminative information between MU action potentials, thereby facilitating higher MU identification^9^.

Our analysis revealed no significant differences in the number of MU detected between young and older adults, likely due to the comparable MED observed in both groups. By contrast, males and females differed significantly in FM% and MED. These differences could reflect sex-specific FM distribution: females tend to accumulate adipose tissue in the gynoid region (i.e., hips, gluteal area, and thighs) due to higher circulating adipokines and hormonal factors, whereas males typically show greater abdominal fat deposit^24^. The higher FM% and MED in the femorogluteal region, which is more prevalent in females, contributed to the reduced number of detected MU from VL compared with males. Nevertheless, including female participants in future research remains essential, as available evidence on sex-specific neural control of movement and adaptations to different stimuli is still limited. This is particularly relevant given recent findings documenting distinct neural behaviors between sexes^33^.

While this study focused on the influence of FM% and anatomical factors, we acknowledge that other variables, related to HDsEMG signal recording also contribute to variability in decomposition outcomes. For instance, the configuration of HDsEMG electrode grids plays a critical role: reducing inter-electrode distance and increasing the number of electrodes improves the MU detection accuracy^34^. For instance, previous research^35^ reported that using four 64-electrode grids to record VL activity during isometric knee extensions resulted in the detection of an average of ~ 42 MU per participant at 30% MVF and ~ 26 MU per participant at 70% MVF in a cohort of young adults. Another important factor is the decomposition algorithm. Blind Source Separation approaches, as used here, are limited in accurately discriminating individual MU action potentials at higher force levels, where the recruitment of additional MU^36^ leads to greater spatial and temporal superimposition (overlap) of action potentials^9,37^. Larger high-threshold MU generate action potentials with greater energy, which can superimpose and obscure those of smaller MU recruited at lower forces^38^, ultimately reducing the number of detectable MU at higher contraction intensities, as also observed in the present study. This overlap complicates the separation of individual MU spike trains, ultimately the number of MU at higher contraction intensities. To address this issue, previous studies have suggested applying more selective spatial filters and increasing the electrode grid density^16,38^.

Despite the methodological strengths of this study, several limitations should be acknowledged. First, skinfold measurements were not included, although they could have provided a more localized estimation of subcutaneous fat. While relatively direct and accessible, their accuracy decreases in individuals with higher adiposity, where pinching thicker fat layers becomes challenging^39^. Second, the sample included relatively few females compared with males. Although the primary aim was not to compare sexes but to identify factors influencing MU detection from VL in a heterogeneous cohort of healthy individuals, the limited number of females may reduce the generalizability of sex-specific findings. Nevertheless, this is the first study to examine the influence of FM% and muscle size on MU detection in both sexes. Lastly, our analysis focused only on the VL muscle, which may limit the generalizability to other muscles. However, these findings extend prior evidence from the biceps brachii^15^, where greater MED and higher force levels similarly posed challenges for MU identification.

In conclusion, our results indicate that both whole-body and regional FM%, together with MED, largely determined by subcutaneous fat within the volume conductor, are negatively associated with the number of detectable MU from VL. For the first time, we identified an average threshold of ~ 0.7 cm MED, below which a greater number of MU can be accurately detected. These results highlight the value of incorporating MED and regional FM% assessments in surface HDsEMG studies, as they may provide important insights into the potential number of detectable MU even before electrode placement. This approach may facilitate the interpretation of decomposition outcomes and support participant recruitment, particularly when aiming to recruit individuals from whom it is more likely to identify a greater and more representative number of MU.

## Methods and materials

### Participants

Thirty-three young adults (age range: 19–30 year.; females: 9) and 28 older adults (age range: 66–82 year.; females: 6) volunteered to take part in this cross-sectional study (Table 1). As general requisites for inclusion, participants had to be between 18 and 35 years for young adults and more than 65 years for older adults. Young female participants were tested during the luteal phase of the menstrual cycle to avoid any potential confounding effects of hormonal changes on MU properties^40^. Exclusion criteria include: (i) history of traumatic lower body injury or surgery, (ii) use of pharmacological treatments that affect the neural response to muscle contractions or alter body fluid balance (e.g., corticosteroids), and (iii) self-reported history of chronic diseases (e.g., diabetes mellitus, neuromuscular disorders, cardiovascular diseases).

The experimental protocols and procedures were approved by the Internal Review Board of the Department of Biomedical Sciences of the University of Padua (HEC-DSB/03–2023) and conformed to the standards set by the *Declaration of Helsinki*. All participants provided written informed consent before their involvement in the study.

### Experimental overview

The study was conducted over a twelve-month period at the Nutrition and Exercise Physiology Laboratory, University of Padua. Participants attended two experimental sessions at the laboratory, separated by at least 48 h.

In the first session (visit 1), participants were informed of the experimental procedures, signed the informed consent form, and completed the validated Italian version of the Global Physical Activity Questionnaire (GPAQ)^41^ to assess habitual physical activity levels. Participants then underwent anthropometric measurements using a portable stadiometer and body composition assessments using BIA, and DXA. Lastly, participants were familiarized with the experimental setup by performing a series of maximal and submaximal isometric voluntary contractions of the knee extensors while seated in a custom-built knee extension dynamometer (see Sect. “Ultrasonography and neuromuscular assessment (visit 2)”).

In the second session (visit 2), an expert operator assessed quadriceps femoris muscle cross-sectional area (CSA), MED, and muscle thickness (MT) using US. Subsequently, participants completed the neuromuscular assessment, which included unilateral maximal voluntary contractions (MVC) and submaximal isometric trapezoidal contractions at 15%, 35%, 50%, and 70% MVF. Concurrently, HDsEMG signals were recorded from the VL muscle.

Participants were instructed to avoid caffeine and energy drinks and to refrain from strenuous physical activity for 24 h prior to each experimental session.

### Experimental procedures

#### Anthropometric and body composition assessment (visit 1)

Body mass and height were measured to the nearest 0.1 kg and 1 cm, respectively, using a portable stadiometer (Wunder, Holtain Ltd., Crymych, UK). BMI was calculated as body mass (kg) divided by height squared (m^2^).

**Fig. 6:**
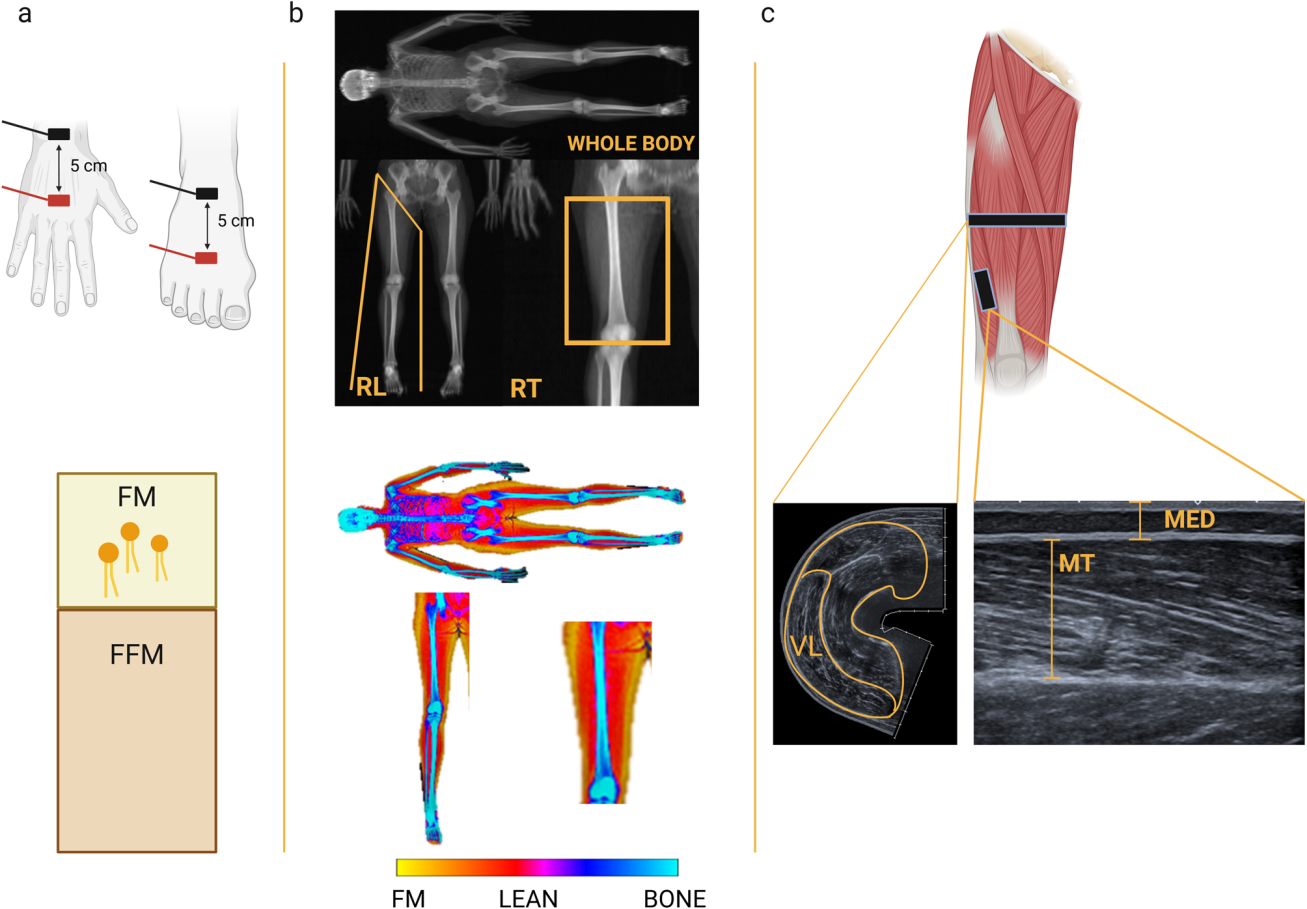
Experimental procedures and data analysis. The upper section illustrates the methodological setting of each device, while the lower section presents the respective outputs. **(a)** BIA (bioelectrical impedance analysis): hand and foot electrode placement. The current electrodes are marked in red, and the voltage electrodes in black. BIA was used to estimate whole-body FM%. **(b)** DXA (dual-energy X-ray absorptiometry) whole-body scan and regional segmentation. **(c)** Ultrasound measurements: the cross-sectional area of the quadriceps was measured at 50% of the femur length. On the left, the quadriceps and vastus lateralis profile are highlighted; the right image displays the longitudinal scan, emphasizing MT (muscle thickness) and MED (muscle-electrodes distance). FM: fat mass; FFM: free-fat mass; RL: right leg, RT: right thigh.

Foot-to-hand BIA was performed with participants lying supine over a non-conductive surface, with lower limbs abducted at 45°, upper limbs abducted at 30° relative to the body midline, and hands pronated^42^. After skin preparation, two adhesive electrodes (Biatrodes Akern Srl, Firenze, Italy) were placed on the dorsal surface of the right foot and two on the dorsal surface of the right hand, spaced 5 cm apart, according to previous studies^42,43^ *(*Fig. 6, a*)*. After a two-minute rest to allow body fluid stabilization, a single frequency of 50 kHz device (BIA 101 BIVA PRO, Akern Systems, Firenze, Italy) was used to measure bioelectrical resistance (Rz) and reactance (Xc) expressed in ohms (Ω).

A whole-body DXA scanner (QDR 4500 W, Hologic Inc., Marlborough, Massachusetts, USA) was used to assess FM% of the whole-body, right leg, and thigh *(*Fig. 6, b*)*. The DXA scanner was calibrated daily according to the manufacturer’s instructions using a standard calibration block (Hologic DXA Quality Control Phantom Lumbar Spine).

In the current study, we combined DXA and BIA to strengthen the reliability of our findings and to provide complementary tools that could also serve as alternatives when one method is not available in a research setting. DXA was selected because it is widely used in research and considered a reference technique for body composition assessment, despite its higher cost and minimal radiation exposure^26^. BIA was included as a practical and accessible method that does not require specialized personnel, is free of radiation, and allows rapid estimation of FM% when validated algorithms are applied^27^. Skinfold thickness measurements were not included because, although cost-effective and informative of subcutaneous fat^44^, they provide less accurate estimates in individuals with higher adiposity, where pinching thicker fat layers is challenging^39,45^.

#### Ultrasonography and neuromuscular assessment (visit 2)

Ultrasound was adopted to obtain local measurements of the quadriceps muscle and VL, with particular focus on MED as an indicator of the thickness of subcutaneous tissue within the volume conductor. This method was prioritized because it provides regional information that cannot be captured by whole-body techniques such as DXA or BIA, and is therefore directly relevant to factors influencing MU detection.

Prior to the neuromuscular assessment, US images of the quadriceps muscle and VL were captured at rest (Xario 100 TUS-X100, Toshiba Medical Systems Corporation, Tochigi, Japan). To assess the CSA of the quadriceps muscle, participants lay supine on a massage bed with their knee joint extended. First, an operator marked 50% of the femur length using a dermatographic pen, defined as the midpoint between the greater trochanter and the lateral mid-patellar point. An experienced operator (MVF) then positioned a linear array transducer (64 mm; PSU-25BT, 12 mHz) transversely on the medial side of the thigh and began acquisition upon identifying the medial borders of the vastus medialis. The scan continued by gradually moving the transducer with minimal pressure applied on the dermal surface in a medial-to-lateral direction until the lateral limits of the VL were identified (Fig. 6, c). Water soluble transmission gel was applied above the muscle to optimize US image detection. Two US images per participant were captured and subsequently used for analysis.

Additionally, two longitudinal (i.e., transducer aligned with the fascicle plane) US images were captured to quantify the MED and MT of VL. MED was used as an indicator of subcutaneous adipose tissue between the muscle belly and the recording electrode grid while MT as an index of VL muscle size *(*Fig. 6, c*)*. During US image capture, participants were seated in the custom-built dynamometer, with their dominant knee (i.e., the right side in all participants) flexed at ~ 115° to replicate the neuromuscular test’s experimental setup. MED and MT were calculated in the area where the electrode grid was later applied (*see* Sect. “Force and HDsEMG signal recordings”) with the skin marked accordingly. The operator applied minimal pressure when positioning the transducer on the skin prior to the acquisition of the images. Two longitudinal US scans parallel to the muscle fibers were acquired, one 1 cm above and one 1 cm below the marked line, to cover the entire area corresponding to the subsequent electrode grid placement.

**Fig. 7:**
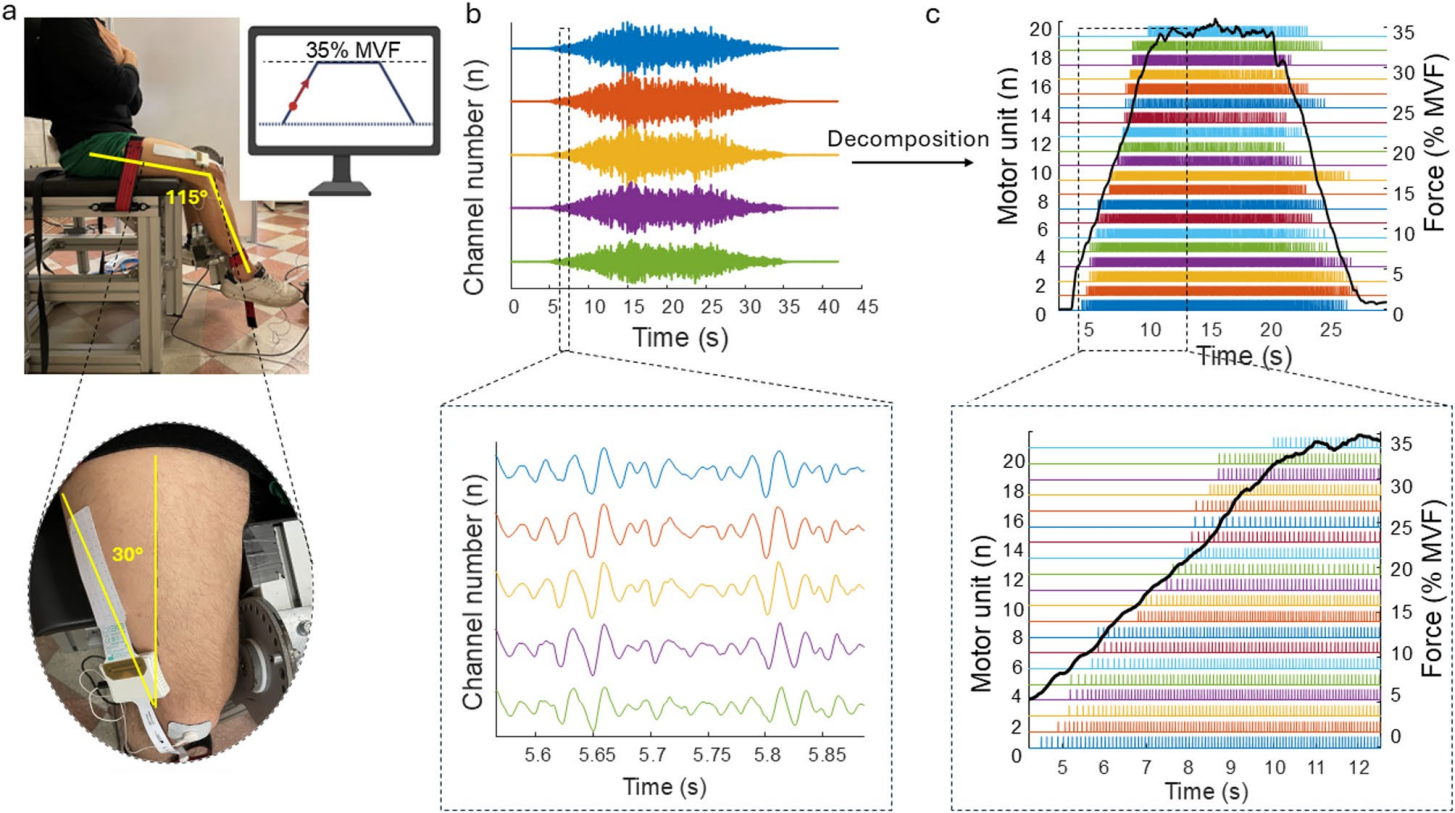
Experimental set-up and HDsEMG data acquisition. **(a)** Participants were seated on a custom-built isometric dynamometer with their dominant knee flexed at 115°. A monitor provided visual feedback during the contractions. A 64-channel HDsEMG grid was placed over the vastus lateralis muscle belly. **(b)** Representative raw signals from five consecutive channels recorded during isometric knee extensions at 35% MVF. The signals were filtered and decomposed into individual motor unit. **(c)** In this participant, a total of 20 motor units were identified. Each colored band represents the discharge times (spike trains) of an individual motor unit, whereas the black line indicates the performed force signal.

After US imaging, the neuromuscular assessment consisted of the simultaneous recording of knee extensors voluntary force and HDsEMG from the VL of the dominant leg. Participants were seated in a rigid custom-built isometric dynamometer, which was adjusted to their height and lower limb length. Specifically, participants’ trunk rested on a rigid backrest with the hip and knee flexed at ~ 100° and ~ 115° (where 180° represents full extension), respectively (Fig. 7, a). This positioning ensured that the dynamometer’s rotational axis aligned with the medial femoral condyle. This specific knee joint angle was adopted in order to maximize the expression of isometric knee extension force output, according to previous studies^46,47^. The ankle of the dominant leg was tightly strapped to an adjustable ankle brace, positioned ~ 3 cm above the malleolus, and in series to a calibrated uniaxial load cell (Kraftaufnehmer Type S9). To prevent any compensation or extraneous movements during the contractions, Velcro straps were fastened at the ankle, thigh, pelvis, and shoulder levels.

Participants first completed a standardized warm-up consisting of submaximal isometric knee extensions (3 × 50%, 3 × 75%, and 1 × 90% of perceived MVF)^15,23^. They then performed three to five MVCs, during which they were verbally encouraged to ‘push as hard as possible’ for 3 to 5 s. The rest between contractions was ~ 60s. The highest instantaneous force recorded during any MVCs was designated as the knee extensor MVF and used as a reference for the subsequent submaximal contractions. After a 5-minute rest, participants performed trapezoidal contractions at four target force levels (2 × 15%, 2 × 35%, 2 × 50%, and 2 × 70% MVF) in a randomized order. Each contraction included a linear ascending phase to the target force, a 10 s plateau phase of constant force production at the target force, and a linear descending phase returning to resting values. The rate of force increase and decrease were held constant at 5% MVF·s^−1^ for all contractions. Participants were instructed to exert force as precisely as possible by matching the visual template of the trapezoidal path displayed on a monitor positioned 1 m in front of them. Trapezoidal contractions were separated by 3 to 5 min of rest. By selecting four distinct target force levels, we were able to identify both low-threshold and high-threshold MU, encompassing nearly the entire range of voluntary recruitment for the VL muscle^17,48^.

#### Force and HDsEMG signal recordings

The analog force signal was amplified (x100), digitized at 2048 Hz using a multichannel amplifier, and sampled in OTBiolab + software (ver. 1.5.7, OT Bioelettronica, Turin, Italy). The same software provided real-time force visual feedback and trapezoidal contraction templates.

The myoelectrical activity was recorded from VL muscle during the submaximal isometric knee extensions using a grid of 64 equally-spaced electrodes (13 rows (10 cm) x 5 (3.5 cm) columns, gold-coated, with a 1 mm diameter and 8 mm interelectrode distance; GR08MM1305, OT Bioelettronica). To position the grid alongside the direction of the muscle fibers, an experienced kinesiologist (AS) marked the skin over the VL at a 30° angle with respect to the line connecting the anterior superior iliac spine and the lateral superior edge of the patella, according to the procedure described in previous studies^23,49^ (Fig. 7, a). Using the US transducer, the same operator adjusted the grid’s angle based on the actual orientation of the muscle fibers. Before electrode placement, the skin surface was shaved, lightly scrubbed with abrasive paste (Everi, SPES Medica, Genoa, Italy) to reduce skin impedance, and cleansed with alcohol wipes. To optimize skin-electrode contact, a disposable adhesive foam layer was applied over the electrodes, with its cavities filled with electroconductive paste (AC Cream SPES Medica, Genoa, Italy), before being attached to the skin. Additionally, hypafix tape was used to secure the electrode grid and prevent detachment during the contractions. Reference electrodes were placed on the patella (HDsEMG grid reference) and on the medial malleolus (main ground electrode) of the dominant leg.

The HDsEMG signals were acquired in monopolar derivation, amplified (x150), sampled at 2048 Hz, and converted to digital data by a multichannel amplifier 16-bit A/D (EMG Quattrocento, OT Bioelettronica, Turin, Italy) before offline analysis. HDsEMG signals were recorded using OTBiolab + software (ver. 1.5.7, OT Bioelettronica, Turin, Italy) and synchronized with the force signal at the source.

### Data analysis

#### Body composition and ultrasound measurements

To estimate the FM% from the BIA assessment, we used the validated prediction equation by Kanellakis and colleagues (2020). This equation estimates the kilograms of fat-free mass based on Rz, Xc, gender, height, and body mass. Consequently, the percentage of fat-free mass was calculated based on each participant’s body mass, and the FM% was subsequently derived from this calculation (Fig. 6, a).

DXA scans were analyzed by the same operator (AS) using the Hologic APEX software (ver. 5.6.0.5) in order to extract data on whole-body FM%, and FM% of the right leg and thigh. Body regions were outlined manually using the tools provided by the software. The right leg was defined as the area bounded superiorly by the right groin line and medially by the vertical line separating the two legs. The right thigh was determined as the area between the lower margin of the ischial tuberosity and the lower margin of the femoral condyles, as previously described^50,51^ (Fig. 6, b).

Another operator (GS) digitally analyzed all the US scans using ImageJ image analysis software (https://imageJ.nih.gov/ij/). From each CSA image, the sizes of the quadriceps muscle and VL only were measured by tracing the muscle borders, excluding the muscular aponeurosis (Fig. 6, c). Two images were analyzed for each variable and the corresponding measurements were averaged to yield a single value for statistical analysis. From the longitudinal US scans (i.e., transducer aligned with the fascicle plane), MT was calculated as the perpendicular distance between superficial and deep aponeuroses of the skeletal muscle in three different points (i.e., proximal, central, and distal portions of the captured image) (Fig. 6, c). The average of the 3 measures of the two captured images was computed, yielding a single value for the statistical analysis. From the same longitudinal US scans, MED was extracted and measured as the perpendicular distance from the surface skin to the superficial portion of the skeletal muscle and included the layers of the skin, subcutaneous fat, and the superficial muscle aponeurosis (Fig. 6, c). For each acquisition, MED was measured at three points (i.e., 1 cm from the start, in the middle, and 1 cm before the end of the image). These measurements were averaged, and the resulting value was further averaged with the values from the other image to produce a single value used for the statistical analysis.

#### Force and HDsEMG signals

The force signal was recorded in millivolt (mV) and converted to Newtons (N). The offset was removed by subtracting baseline force values generated by the weight of the leg against the load cell. The signal was then filtered with a 4th -order, zero-lag Butterworth filter with a cut-off frequency of 15 Hz. The acquired force signal was processed offline using Matlab software (ver. 2023b, The MathWorks, Natick, MA, USA). MVT was calculated as the product of MVF and moment arm length (i.e., the distance between the lateral femoral condyle and the load cell), to allow direct comparison with previous studies.

In offline analysis, the HDsEMG signals were band-pass filtered (20–500 Hz, 2nd order, zero-lag Butterworth filter) (Fig. 7, b). The filtered signals from individual submaximal contractions were decomposed into individual MU spike trains using the Convolution Kernel Compensation algorithm implemented in the DEMUSE tool software^4,29^, which is known for its accuracy, reliability and validity for single MU identification over a broad range of voluntary force contractions^29^. The accuracy of the decomposition outcome was determined by calculating the pulse-to-noise ratio (PNR) for each MU. The identified MU spike trains were visually inspected and manually edited by an experienced investigator according to available guidelines^9^. Only the MU with PNR > 28 dB (sensitivity of > 85%) were considered for further analysis^5,29^ (Fig. 7, c). All the recordings were decomposed and visually inspected; however, for the statistical analysis, we selected the trial with the highest number of accurately identified MU at the same force target. The number of identified MU was then used as the outcome measure for subsequent analysis.

### Statistical analysis

The Shapiro-Wilk test was used to assess the normality of the data, while the Levene’s test was applied to evaluate the homogeneity of variance. Since most variables were not normally distributed or did not meet the assumption of equal, non-parametric tests were adopted.

To assess differences in body composition, muscle force, physical activity levels, and the number of identifiable MU from VL, Mann-Whitney U tests were performed between groups (young vs. older adults; females vs. males). Differences in the number of MU identified at the target forces across the whole cohort were assessed using Friedman’s test, followed by Conover’s post hoc test with Bonferroni correction when a significant main effect was observed. Spearman’s rank correlations were computed to examine the associations between the number of identifiable MU from the VL and whole-body and regional FM%, MED, MT, and CSA. Statistical significance was set at *p* < 0.05. All analyses were performed using the software package JASP for Windows (JASP Team, 2024; version 0.18.3).

Given that MED showed the strongest correlation with the number of detectable MU additional analyses were conducted to explore the presence of a potential threshold. First, we a quartile-based approach was adopted by dividing participants into quartiles (Q1-Q4) according to their MED values. Group comparisons were performed to assess differences in the average number of MU across quartiles using the Kruskal-Wallis test, followed by Dunn’s post hoc test with Bonferroni correction when a significant main effect was detected. Subsequently, segmented regression analysis was performed between MED and the number of identified MU using the “segmented package” in R software (version 2025.09.0). This analysis was applied both to the average number of MU identified across all contraction intensities and separately for each intensity level. For all models, slope coefficient estimates and the 95% confidence intervals (CIs) of the estimated thresholds were computed.

## Data Availability

The datasets used and analysed during the current study are available from the corresponding author on reasonable request.

## Abbreviations

BIA: Bioelectrical Impedance Analysis
BMI: Body Mass Index
CSA: Cross-Sectional Area
DXA: Dual-energy X-ray Absorptiometry
EMG: Electromyography
FM%: Relative Fat Mass
GPAQ: Global Physical Activity Questionnaire
HDsEMG: High-Density surface Electromyography
MED: Muscle-Electrode Distance
MT: Muscle Thickness
MU: Motor Units
MVC: Maximal Voluntary Contraction
MVF: Maximal Voluntary Force
MVT: Maximal Voluntary Torque
PNR: Pulse-to-Noise Ratio
US: Ultrasound
VL: Vastus Lateralis
